# Lesion size affects the risk of technical difficulty in gastric endoscopic submucosal dissection

**DOI:** 10.1038/s41598-024-52150-z

**Published:** 2024-01-17

**Authors:** Yuqi Zhao, Xiaogao Pan, Yihan Chen, Yuyong Tan, Deliang Liu

**Affiliations:** 1 Department of Gastroenterology, Second Xiangya Hospital, Central South University, 139 Renmin Road, Changsha, 410011 Hunan Province China; 2 Research Center of Digestive Disease, Central South University, 139 Renmin Road, Changsha, 410011 Hunan Province China; 3 Clinical Research Center for Digestive Disease in Hunan Province, 139 Renmin Road, Changsha, 410011 Hunan Province China; 4 Department of Emergency Medicine, Second Xiangya Hospital, Central South University, Changsha, China; 5 Emergency Medicine and Difficult Diseases Institute, Central South University, Changsha, China

**Keywords:** Gastric cancer, Risk factors

## Abstract

Current evidence shows an inter-country inconsistency in the effect of lesion size on the technical difficulty of gastric endoscopic submucosal dissection (ESD). We aimed to evaluate the specific correlation and quantify the ensuing risks. This retrospective study consisted of 405 ESD cases with gastric single lesion from April 2015 to April 2023. The correlation and risk prediction of lesion size with technical difficulty was explored to provide further clinical evidence. An additive generalized model and recursive algorithm were used to describe the non-linear association, and a linear two-piece regression was constructed to analyze the inflection point. Subgroup analysis and interaction were used to explore intergroup characteristics. Overall, difficult cases had larger lesion sizes, and the more significant the increase, the higher the risk of technical difficulty. In the full model, after adjusting for all covariates, each 1 mm, 3 mm, 5 mm, 7 mm, and one standard increase in lesion size increased the risk of technical difficulty by 8%, 26%, 42%, 72%, and 125%, respectively. There is a nonlinear positive correlation between lesion size and risk of technical difficulty, and the premeditated inflection point was 40 (mm) via two-piecewise linear regression and recursive algorithm. Subgroup analysis showed a stronger correlation between lesion size and difficult ESD in the upper site and submucosal fibrosis groups. Available evidence suggests that lesion size as a risk signal nonlinearly increases the technical difficulty of gastric ESD procedure, especially in cases of upper site and submucosal fibrosis, which deserves further investigation.

## Introduction

Endoscopic submucosal dissection (ESD) was first reported in 1988 as a promising technique for the en bloc removal of dysplastic and early cancer lesions throughout the gastrointestinal tract^1,2^. Developed to date, ESD has become a well-established procedure to resect large superficial gastric lesions with high rates of en bloc resection, few recurrences, and favorable long-term outcomes^3^. Nevertheless, it has been recognized as essential that appropriate training and sufficient opportunities are vital settings to improve technical skills^4^. ESD is technically challenging and demanding with a steep learning curve for clinical endoscopists. Evaluating the risk of technical difficulty before the procedure is crucial to improve the quality of gastric ESD training and outcomes.

In recent years, increasing efforts have been made to assess the predictors of difficult ESD, including lesion size, location, appearance, and invasion depth^5–7^. These efforts, while useful, appeared to be partly controversial regarding the influence of lesion size in North America^1^. We did notice that lesion size in clinical practice tended to increase the technical difficulty, such as perforation, bleeding, incomplete resection, and prolonged procedure duration. However, there remains an absence of research on the specific correlation (linear or nonlinear) and the consequent risk prediction. Hence, based on our center's experience, we attempt to assess the effect and interaction of lesion size with the risk of technical difficulty in gastric ESD to provide further clinical evidence.

## Materials and methods

### Patients and data collection

**Figure 1:**
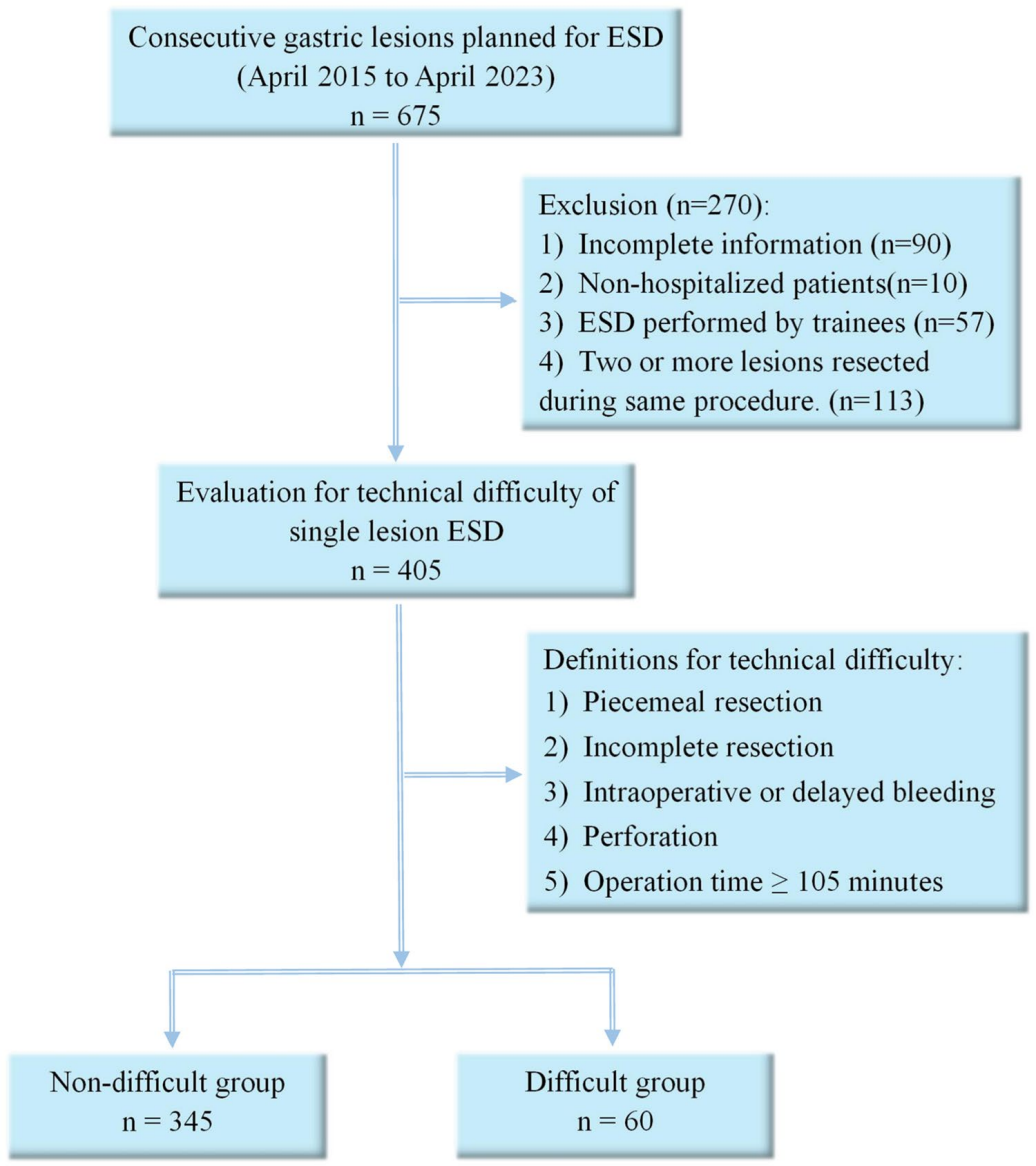
Flow chart of patient enrollment. ESD, endoscopic submucosal dissection.

A total of 675 cases received gastric lesion ESD in our hospital during April 2015 to April 2023. All patients were informed about the risks and benefits and provided written informed consent preoperatively. We non-selectively and consecutively collected data at our hospital. The following were exclusion criteria: 1) incomplete information; 2) non-hospitalized patients; 3) ESD performed by trainees due to inexperience (≥ 50 ESDs (total) were considered experienced); 4) two or more lesions resected during the same procedure (Fig. 1)^4,8^. Based on these criteria, 405 cases with single lesion ESD were enrolled for the assessment.

Anonymous data were compiled from the hospital's electronic medical record system. The data collection and analysis followed the Ethics Committee of the institution and the Declaration of Helsinki. The study protocol was approved by the Ethics Review Board of the Second Xiangya Hospital (Central South University; No. 2020K014).

The medical records and endoscopic findings were carefully reviewed to collect patient demographics (age, gender), lesion characteristics (histology, location, gross type, surface configuration, submucosal fibrosis, invasion depth), procedure-related factors and clinical outcomes, including en bloc resection, histologically complete resection and the presence of adverse events.

### Endoscopic equipment and accessories

The ESD procedures were performed under general anesthesia with endotracheal intubation. A single-channel endoscope (GIF-Q260J; Olympus Corp., Tokyo, Japan) was used, with a transparent cap (D-201–11,802, Olympus) attached to the front. A carbon dioxide insufflator (UCR; Olympus) was used. Other equipment and accessories used during the ESD procedure included a high-frequency generator (ICC 200; ERBE Elektromedizin GmbH, Tübingen, Germany), a dual knife (KD-650L, Olympus), an insulation-tipped knife (IT-Knife, KD-611L, Olympus), an argon plasma coagulation unit (APC300; ERBE), and an injection needle (NM-4L-1; Olympus). A solution consisting of 100 mL saline + 5 mL 0.2% indigo carmine + 1 mg epinephrine was used for submucosal injection during the ESD procedure.

### Gastric ESD procedure

**Figure 2:**
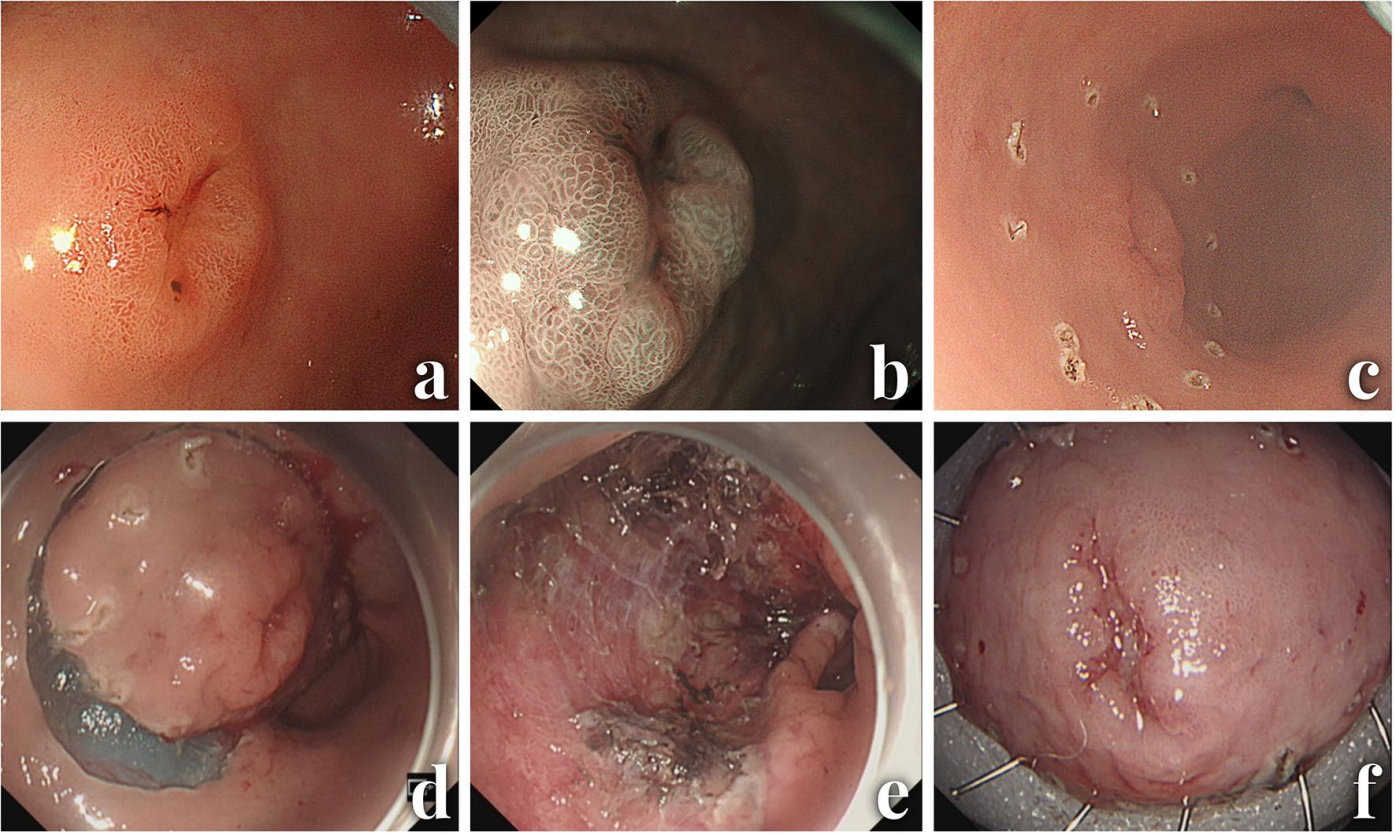
Endoscopic submucosal dissection procedure. (**a**) Endoscopic view of a lesion located at the lower third of the stomach. (**b**) Narrow band imaging with magnifying endoscopy image. (**c**) Mark the edge of the lesion. (**d**) Peel off the lesion along the marking line. (**e**) The wound surface after ESD. (**f**) The resected specimen was stretched and pinned down with needles.

The absolute and expanded criteria of endoscopic therapy and detailed ESD procedures have been widely described^9–11^. To summarize, the protocol consists of 5 steps: (1) marking dots around the lesion; (2) submucosal injection of a solution; (3) mucosal incision with a dual knife and submucosal dissection with a dual knife in epithelial lesions or an IT knife in non-epithelial lesions; (4) coagulating visible vessels; and (5) retrieval of the specimen (Fig. 2). All ESD procedures were performed by certified endoscopists with sufficient expertise. No assisting methods or techniques were used during the study period.

### Histologic evaluation

All ESD resected tissue specimens were stretched and pinned down with needles after removal, fixed in 10% formalin solution, and assessed microscopically. The histology diagnosis was based on the World Health Organization (WHO) classification for tumors of the digestive system^12^.

### Definitions

The primary outcomes were the proportions of en bloc and complete resection, and the ESD-related adverse events including intraoperative bleeding and perforation. En bloc resection is defined as the excision of the visible targeted lesion in a single specimen. Complete resection (R0) is defined as en bloc resection with lateral and deep margins free of neoplasia on histologic evaluation. In contrast, incomplete resection (R1) is infiltrated tumor margins or undetermined margins due to coagulation artifacts or piecemeal resection^13^. Intraoperative bleeding refers to active oozing or jet bleeding during the procedure, which is difficult to stop endoscopically. It is necessary to discontinue the procedure and/or transfusion therapy with a 20 g/L reduction in hemoglobin concentration compared to the preoperative period^13^. The presence of perforation can be confirmed by endoscopy during the procedure or by free air in the subphrenic space found on an upright x-ray or computed tomography scan after the procedure^6,13^.

The classification of lesion gross type is based on the Paris standard: 0-IIa, 0-I types are defined as elevated lesions, 0-IIb types are defined as flat lesions and 0-IIc, 0-III types are defined as depressed lesions^14,15^. The gastric lesion location is divided into three categories: upper third, middle third, and lower third^13^. Submucosal fibrosis is defined as the presence of lesions with scarring from previous ulceration or visible fibrosis identified during dissection^13^. Invasion depth was based on preoperative endoscopic ultrasonography, intraoperative findings, and postoperative histology for final confirming the involved layer^13^. The procedure time is calculated from entering the endoscopy to withdrawing the endoscopy. The lesion size is denoted by the largest diameter after pinning by needles.

### Technical difficulty endpoints

Based on previous studies and our center's experience, we attempted to define technically difficult gastric ESD as having at least any of the following endpoints: piecemeal resection, R1 resection, intraoperative bleeding, perforation, and procedure time ≥ 105 min^5,6,10,16^. In general, piecemeal resection, R1 resection, and adverse events are the main factors reflecting the technical difficulty in the ESD procedure, and prolonged operation duration is also an important sign. Due to more considerations, additional device exchanges, and more bleeding, several studies have used prolonged procedure time as an endpoint to define technically difficult ESD^5,6,16^. We set 105 min as the threshold for the following reasons: (1) 105 min is very close to the mean plus the standard deviation of the procedure time in our study (103.96 min); (2) in daily practice, manipulation duration within 105 min might be acceptable for the majority of patients, from entering the endoscope to withdrawing the endoscope.

### Statistical analysis

Continuous variables were expressed as mean ± standard deviation. Categorical variables were shown as percentages. Kruskal–Wallis test (skewed distribution), chi-squared test (categorical variables), or ANOVA (one way) were used to analyze normally distributed data. Statistical analyses were performed in three steps to investigate whether lesion size correlated with technical difficulty for certain members^17^.

In step 1, Least Absolute Shrinkage and Selection Operator (LASSO) regression was applied to minimize the potential collinearity and over-fitting of variables^18^, which was performed to identify the predictors of technical difficulty. Then, three covariates adjustment models were constructed using Logistic regression to show the associated risks.

In step 2, non-linearity in lesion size and technical difficulty was addressed. The fitting of an additive generalized model and penalized spline method (smooth curve) was done. In case any detection of non-linearity was observed, the point of inflection was calculated using a recursive algorithm, and a linear two-piece regression was constructed. This was done on the inflection point for both sides. For the likelihood log-ratio test, the best-fit model was checked on the P-values.

In step 3, a stratified linear regression model was used for subgroup analyses and interaction. Continuous variables were changed to categorical variables (T1, T2, T3) as stated in the clinical tertile (cut point). A sensitivity analysis was used to confirm the stoutness of data analysis that converted lesion size to a categorical variable and the trend’s p-value was calculated, which aimed to detect the likelihood of non-linearity.

EmpowerStats (http://www.empowerstats.com, X&Y Inc Solutions, Boston, MA) and R version 4.0.5 (http://www.r-project.org) were used for statistical analyses. E-values were calculated using the *E* value package in R to provide an estimate of the effect size required for unmeasured confounders to explain the observed association^19^. P ≤ 0.05 was considered statistically significant.

## Results

### Characteristics of baseline

The 405 patients with gastric single lesions had a mean age of 50.37 ± 11.13 years, 214 (52.84%) were female, and 60 (14.81%) cases were technically difficult. The mean resected lesion size was 19.61 ± 10.44 mm, and the mean ESD procedure time was 61.09 ± 32.28 min. The differences in baseline characteristics were presented in Table 1. Compared with the non-difficult group, difficult cases seemed to have larger lesion sizes and had a higher probability in the groups: elder, histological diagnosis as high-grade neoplasia, lesion of flat type, erythema, submucosal fibrosis, and lesion from submucosa or muscularis propria. No statistically significant differences were observed for gender, other histological diagnoses, lesion location, other gross types, and other surface configurations (P > 0.05). Baseline characteristics of the patients based on the tertile lesion size were also shown in Supplementary Table S1. The uppermost group of lesion size (T3) had higher values in age and procedure time, and incidence of difficult ESD. The comparison of lesion size stratified by difficult ESD was shown in Supplementary Fig. S1.

**Table 1:** Baseline characteristics and comparison between non-difficult ESD and difficult ESD.

Characteristics	Total	Non-difficult ESD	Difficult ESD	P-value
No. patients	405	345	60	
Age, year	50.37 ± 11.13	49.11 ± 12.35	59.41 ± 8.25	< 0.001
< 60	298 (73.58%)	274 (79.44%)	24 (40.00%)	< 0.001
≥ 60	107 (26.42%)	71 (20.56%)	36 (60.00%)	< 0.001
Gender				
Male	191 (47.16%)	163 (47.25%)	29 (48.33%)	0.951
Female	214 (52.84%)	182 (52.75%)	31 (51.67%)	
Histology				
Epithelial lesions	251 (61.97%)	210 (60.87%)	41 (68.33%)	0.306
Negative for neoplasia	98 (24.20%)	90 (26.08%)	8 (13.33%)	0.101
Indefinite for dysplasia	6 (1.48%)	6 (1.74%)	0 (0.00%)	0.401
Low-grade dysplasia	66 (16.30%)	56 (16.23%)	10 (16.67%)	0.929
High-grade dysplasia	80 (19.75%)	58 (16.81%)	22 (36.67%)	0.005
Invasive neoplasia	1 (0.25%)	0 (0.00%)	1 (1.67%)	0.198
Non-epithelial lesions	154 (38.02%)	135 (39.13%)	19 (31.67%)	0.306
Location				
Upper third	60 (14.81%)	53 (15.36%)	7 (11.67%)	0.268
Middle third	56 (13.83%)	41 (11.89%)	15 (25.00%)	0.096
Lower third	289 (71.36%)	251 (72.75%)	38 (63.33%)	0.163
Gross type				
Elevated	360 (89.89%)	311 (90.14%)	49 (81.67%)	0.095
Flat	35 (8.64%)	26 (7.54%)	9 (15.00%)	0.010
Depressed	10 (2.47%)	8 (2.32%)	2 (3.33%)	0.396
Surface configuration				
Erythema	70 (17.28%)	46 (13.22%)	24 (40.00%)	< 0.001
Ulcer	53 (13.09%)	43 (12.46%)	10 (16.67%)	0.448
Nodularity	43 (10.62%)	31 (8.99%)	12 (20.00%)	0.043
Submucosal fibrosis				0.038
No	384 (94.81%)	331 (95.94%)	53 (88.33%)	
Yes	21 (5.19%)	14 (4.06%)	7 (11.67%)	
Invasion depth				< 0.001
Mucosa	248 (61.23%)	227 (65.80%)	21 (35.00%)	
Non-mucosa	157 (38.77%)	118 (34.20%)	39 (65.00%)	
Procedure time, min	61.09 ± 32.28	50.84 ± 22.67	133.00 ± 40.38	< 0.001
Lesion size, mm	19.61 ± 10.44	17.97 ± 9.15	29.05 ± 12.36	< 0.001

Data are presented as n (%) or mean ± standard deviation.

*ESD* endoscopic submucosal dissection.

The *p* < 0.05 is considered to be statistically significant.

### Adjusted and unadjusted models

All variables measured at the hospital were included in the LASSO regression. After LASSO regression selection (Fig. S2 in the Supplement), nine variables remained significant predictors of technically difficult gastric ESD, including age (≥ 60), lesion of flat type (gross type), upper third (lesion location), erythema, ulcer, nodularity, submucosal fibrosis, lesion from submucosa or muscularis propria (invasion depth), and lesion size.

We defined the above eight variables (except lesion size) as covariates affecting the technical difficulty and constructed three models to analyze the independent effects (univariate and multivariate) based on the Logistic regression model. The odds ratio (OR) and 95% confidence intervals were listed in Table 2. In the full model (Model III), after adjusting for all covariates, for every increment of 1 mm, 3 mm, 5 mm, 7 mm, and one standard in lesion size, the risk of technical difficulty increased respectively by 8%(1.08, 95% CI 1.04–1.13, E-value 1.37), 26% (1.26, 95% CI 1.20–1.81, E-value 1.83), 42% (1.42, 95% CI 1.22–1.66, E-value 2.19), 72% (1.72, 95% CI 1.29–2.30, E-value 2.83), and 125%(2.25, 95% CI 1.46–3.46, E-value 3.93).

**Table 2:** Relationship between lesion size and risk of difficult ESD in different models.

Exposure	Mode I (OR, 95% CI, P)	Mode II (OR, 95% CI, P)	Mode III (OR, 95% CI, P)	E-value
Lesion size, mm (per 1 increment)	1.09 (1.05, 1.12), < 0.001	1.07 (1.04, 1.11), < 0.001	1.08 (1.04, 1.13), 0.000	1.37
Lesion size, mm (per 3 increment)	1.28 (1.17, 1.41), < 0.001	1.23 (1.12, 1.36), < 0.001	1.26 (1.20, 1.81), 0.000	1.83
Lesion size, mm (per 5 increment)	1.52 (1.30, 1.77), < 0.001	1.42 (1.21, 1.66), < 0.001	1.42(1.22, 1.66), 0.000	2.19
Lesion size, mm (per 7 increment)	1.79 (1.45, 2.22), < 0.001	1.63 (1.31, 2.04), < 0.001	1.72 (1.29, 2.30), 0.000	2.83
Lesion size, mm (per SD increment)	2.39 (1.74, 3.28), < 0.001	2.08 (1.49, 2.90), < 0.001	2.25 (1.46, 3.46), 0.000	3.93
Lesion size (tertiles)				
T1 (4–15 mm)	Ref	Ref	Ref	1.00
T2 (15–25 mm)	4.39 (0.92, 21.06), 0.064	3.67 (0.74, 18.17), 0.111	5.21 (0.92, 29.53), 0.062	9.89
T3 (25–70 mm)	2.85 (0.52, 15.47), 0.226	1.60 (0.27, 9.31), 0.602	2.05 (0.29, 15.29), 0.484	3.52
P for trend	< 0.001	0.010	0.014	

Model I adjusted for age and gender. Model II adjusted for Model I + lesion of flat type (gross type), upper third (lesion location), erythema, ulcer, nodularity, submucosal fibrosis, lesion from submucosa or muscularis propria (invasion depth). Model III adjusted for Model I + location (upper, middle, and lower), gross type (elevated, flat, and depressed), surface configuration (erythema, ulcer, and nodularity), submucosal fibrosis (no and yes), and invasion depth (mucosa and non-mucosa). E-value provides an estimate of the required effect size needed for an unmeasured confounder of Model III. T1, T2, and T3 are categorical variables for lesion size, which were transformed from continuous variables with clinical tertiles as cut-off points and used in sensitivity analyses to detect the likelihood of non-linearity.

*ESD* endoscopic submucosal dissection. *SD* standard deviation.

E-value provided an estimate of the required effect size needed for an unmeasured confounder to explain away this association, reinforcing the robustness of the results. However, we also converted lesion size from a continuous variable to a categorical variable (tertiles, T1, T2, and T3). When the lesion size enters the fully-adjusted model as a categorical variable, the trend of the effective value in the different groups is non-equidistant. Based on these non-equidistant changes in effects, there may be a non-linear relationship between lesion size and risk of technically difficult gastric ESD.

### Non-linear relationships

**Figure 3:**
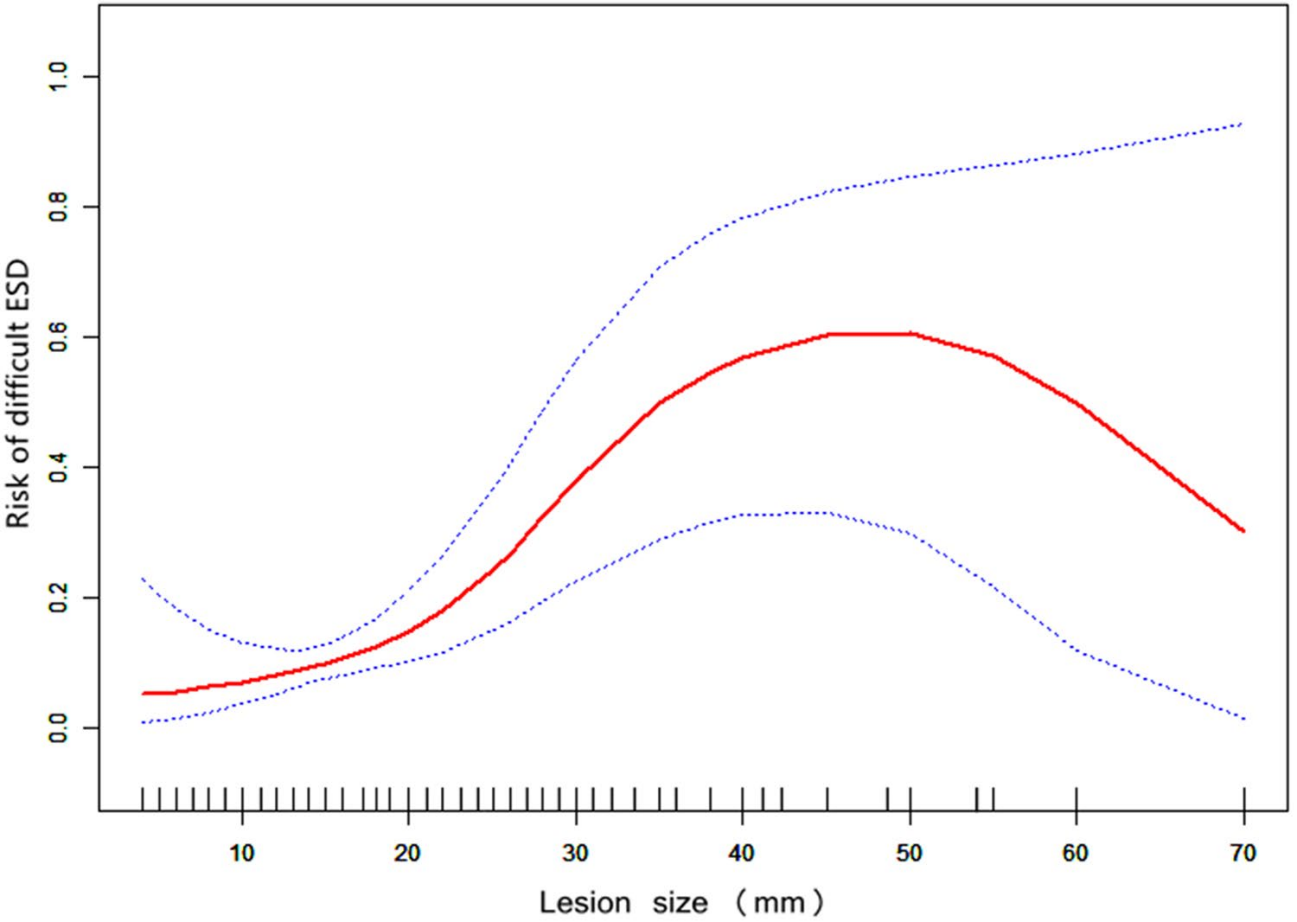
The relationship between lesion size and the risk of technical difficulty. A non-linear association between lesion size and the risk of technical difficulty was found in a generalized additive model (GAM). The solid red line represents the smooth curve fit between variables. Blue bands represent the 95% confidence interval from the fit. All adjusted for age, gender, location (upper, middle, and lower), gross type (elevated, flat, and depressed), surface configuration (erythema, ulcer, and nodularity), submucosal fibrosis (no and yes), and invasion depth (mucosa and non-mucosa).

We evaluated the non-linear correlation between lesion size and the risk of technical difficulty via fitting an additive generalized model and penalized spline method (Fig. 3; Table 3). The smooth curve results revealed the nonlinearly positively correlated after adjusting all covariates. The linear regression model and two-piecewise linear regression model were respectively used to fit the association between lesion size and technical difficulty. The p-value for the log-likelihood ratio test was 0.014, which indicated dual piecewise linear regression was more appropriate due to the perfect representation. The premeditated inflection point was 40 (mm) via two-piecewise linear regression and recursive algorithm. On the right side of the inflection point (≥ 40 mm), the OR and 95% CI were 0.95 and 0.85–1.06, without statistically significant (P > 0.05). On the left side of the inflection point (< 40 mm), the OR and 95% CI were 1.13 (1.06–1.20), which was statistically significant (P < 0.001).

**Table 3:** The results of the two-piecewise linear model.

Exposure	Difficult ESD (OR, 95% CI)	P-value
Fitting model by standard linear regression	1.07 (1.03, 1.12)	< 0.001
Fitting model by two-piecewise linear regression		
The inflection point of lesion size (mm)	40	
< 40	1.13 (1.06, 1.20)	< 0.001
≥ 40	0.95 (0.85, 1.06)	0.339
Difference in two-piecewise effect	0.85 (0.73, 0.98)	0.028
P for log-likelihood ratio test	0.027	

Adjusted: age, gender, location (upper, middle, and lower), gross type (elevated, flat, and depressed), surface configuration (erythema, ulcer, and nodularity), submucosal fibrosis (no and yes), and invasion depth (mucosa and non-mucosa).

*ESD* endoscopic submucosal dissection, *CI* confidence interval, *OR* odds ratio.

The *p* < 0.05 is considered to be statistically significant.

### Subgroup analysis and interaction

**Figure 4:**
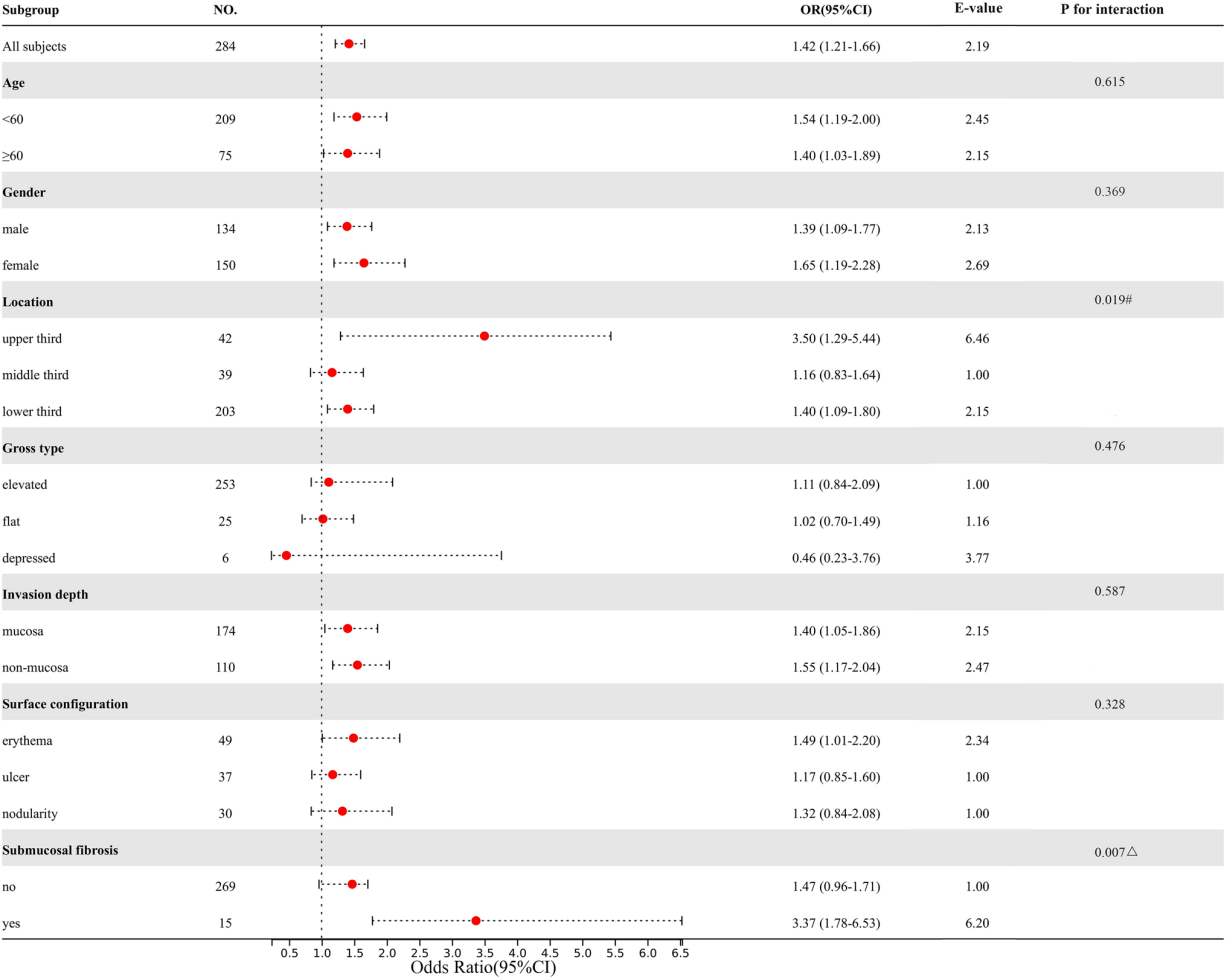
Results of subgroup analysis and interaction analysis between lesion size and the risk of technical difficulty (lesion size per 5 mm increments). All adjusted for age, gender, location (upper, middle, and lower), gross type (elevated, flat, and depressed), surface configuration (erythema, ulcer, and nodularity), submucosal fibrosis (no and yes), and invasion depth (mucosa and non-mucosa). E-value provides an estimate of the required effect size needed for an unmeasured confounder. # P value for the upper third is for the comparison of the middle and lower third. ΔP value for the submucosal fibrosis is for the comparison of non-submucosal fibrosis.

According to clinical guidelines and previous studies, the predetermined covariates were age (< 60 years vs. ≥ 60 years), gender (male vs. female), location (upper vs. middle vs. lower), gross type (elevated vs. flat vs. depressed), invasion depth (mucosa vs. non-mucosa), surface configuration (erythema vs. ulcer vs. nodularity), and submucosal fibrosis (no vs. yes). We examined the interactions between these factors and lesion size (per 5 mm) with a stepwise procedure for multivariate analysis (Fig. 4). The results suggested that the effects in these groups were consistent and stable: age, gender, and invasion depth (all p for interaction > 0.05); and the effects in these groups were not statistically significant: gross type and surface configuration; and the deviations in these groups were more pronounced: upper (vs. middle and lower), and submucosal fibrosis (vs. no) (all p for interaction < 0.05). The baseline characteristics of subgroups were shown in Supplementary Materials Table S2 and Table S3, based on the lesion location and submucosal fibrosis.

## Discussion

Improvements in techniques, refinements in devices, and increased expertise of endoscopists have reduced the overall incidence of adverse events linked to ESD^20,21^. However, limitations such as demanding training, technical difficulty, and longer procedure time are still major concerns regarding this procedure^4^. In clinical practice, technical challenges vary according to patient and lesion characteristics. The key to completing an ESD procedure is to predict the risk of technical difficulty^3^. In our study, we investigated whether lesion size correlated with technical difficulty in those members undergone gastric ESD via three statistical steps. These findings were summarized as follows: (1) Overall, difficult cases had a larger lesion size, and the more significant the increase, the higher the risk of technical difficulty. (2) The lesion size nonlinearly increased the risk of technical difficulty, and the premeditated inflection point was 40 (mm) via two-piecewise linear regression and recursive algorithm. (3) Subgroup analysis showed a stronger correlation between lesion size and difficult ESD in the group with the upper site and submucosal fibrosis.

Since the advent of ESD, there have been numerous reports regarding risk factors associated with technical difficulty. Imagawa et al. reported that larger tumor size, location, and ulceration were related to the greater difficulty of gastric ESD^6^. Kim et al. found that larger lesion size, location at the upper, submucosal fibrosis, and submucosal infiltration were independent risk factors for increasing the technical difficulty of gastric ESD^5^. Bang et al. established a machine learning model to accurately predict the curative resection of undifferentiated type of early gastric cancer before ESD and found that lesion size was the most important feature in each explainable artificial intelligence analysis^22^. Lesion characteristics, especially lesion size, seem to be strong predictors of difficult gastric ESD in these reports on the Oriental population (Japan and Korea). There was a similar conclusion that was confirmed in a multicenter ESD study in Germany by Fleischmann et al.^23^. However, a large prospective multicenter study by Draganov et al. in North America showed that the presence of severe submucosal fibrosis was the strongest predictor for failed ESD. While conversely, neither lesion size nor morphology was shown to impact ESD resection outcomes^1^. These findings appeared to cause some controversy about the influence of lesion size on technical difficulty.

In our findings, we did observe a similar phenomenon linked to Oriental gastric ESD, that is, larger lesion size increased the procedure duration and the incidence of incomplete resection and adverse events. We speculate that this inter-country variation finding results from an imbalance in technological development and skills training^21,24,25^. ESD, which appeared more than two decades ago in Japan, has the first indigenous application advantages in Eastern Asia^2^. Subsequently, European endoscopists have increasingly embraced the therapeutic possibilities offered by ESD^4^, but the adoption of ESD in North America has been cautious and lagged^1^. Perhaps this phenomenon is related to the difference in the incidence of early gastric cancer between eastern and western countries^24,26^. Another important factor is that two or more lesions in these studies are resected during the same procedure, which would increase the endoscopist's fatigue and susceptibility to perforation or bleeding, as well as increase the incidence of adverse cardiovascular events by prolonging anesthesia duration^11,27,28^. Based on these concerns, our study excluded cases with the non-single lesion to more rigorously analyze the relationship between lesion size and technical difficulty.

We observed the non-linearity correlation by fitting the smooth curve and calculated the inflection point as 40 mm using a recursive algorithm. Although there was a weak positive correlation on the right side of the inflection point with unclear statistical significance and even a negative correlation after sizes > 50 mm, this does not diminish the risk warning of technical difficulty with large-sized lesions in practice given the small sample size (n = 22). It is because most large-sized lesions are no longer suitable for ESD treatment owing to their risk of malignant metastasis, resulting in biased data for large-sized lesions with less information. This result may reflect the limitations of a retrospective study and may need to increase the sample size to reduce bias in future studies. Endoscopists still need careful consideration of each step during incision and dissection in large lesion ESD procedure, and thorough tissue elevation and coagulating visible vessels to reduce perforation and bleeding.

Subgroup analysis is important evidence for scientific research to reveal intergroup characteristics^29^. In this study, we found a strong correlation between these groups: upper site and submucosal fibrosis. According to previous reports, patients with these characteristics are prone to incomplete resection and adverse events and are time-consuming for incision and dissection^1,5,6^. Combined with practical experience, we found that controlling the endoscope is more difficult in the upper part of the stomach, and the lesions in the upper site are also prone to arterial bleeding, especially on the side of the lesser curvature, which is supplied predominantly by the right gastric artery. As the pooled blood continuously disturbs the endoscopic hemostasis, it is difficult to obtain clear endoscopic vision to properly use endoscopic equipment^6^. Additionally, the proximal gastric wall is thinner than that of the antrum, and the upper lesions also easy to increase the gastric perforation rate^27,30^. Therefore, it is recommended by experts that beginners perform training in ESD on small lesions of the antrum to increase their expertise^4^. Submucosal fibrosis, accompanied by ulceration scarring or fibrotic adhesions, frequently diminishes the effect of elevation and buffering of submucosal fluid cushion, which predisposes to incomplete resection and gastric perforation during the procedure^8,23^. As expected, lesion size, an independent risk factor, was consistent for patients who were elder or younger and male or female. This result suggests that the risk of technical difficulty with larger lesion sizes does not vary with age and gender, and that vigilance is needed in any case to deal with possible emergencies. Despite the insignificant interaction, we still observed a slightly higher risk of submucosal invasion than the mucosal layer. This is theoretically consistent with expectations, but further evidence is needed^5,31^.

There are several inherent limitations in this retrospective study. First, the ESDs performed by trainees were not included in the present study. A large-scale prospective multicenter study is needed to confirm the generalizability of the performance experience. Second, since no assistive techniques were used in this study, as well as the fact that all epithelial lesions were uniformly treated with a dual knife, and non-epithelial lesions were treated with a dual + IT knife, we were unable to compare the different outcomes of knives and assistive techniques. Third, due to the sample size limitation, lesion size > 40 (mm) in this study showed a weak positive correlation without statistical significance, which needs further research to verify the correlation. Finally, the lesion size in this study was a post hoc analysis obtained from resected lesion measurement. Therefore, it is preferable to use lesion size measured under immediate endoscopy to predict risk, such as a virtual scale endoscope^32^. Unfortunately, there is no standard method to measure lesion size endoscopically, which requires future studies to focus on addressing it.

In conclusion, this may be the first report using lesion size to measure the risk of ESD technical difficulty. These findings in our study may provide some insight that patients with larger lesion sizes may be at higher risk of technical difficulty during ESD, especially on the upper side and accompanied by submucosal fibrosis. In the treatment strategy of these patients, the endoscopists need to consider more details of incision and dissection and, if necessary, use assisting techniques such as traction to ensure the quality of outcomes. This accordingly quantified risk may psychologically alert the endoscopists to improve the procedure schedule and better contribute to a preoperative consensus about the procedural risk of ESD between endoscopists and patients. Although current evidence indicates that this concomitant effect may not simply be linearly superimposed, it is reasonable to assume that lesion size is a strong risk signal for the technical difficulty of ESD, which deserves further study.

### Supplementary Information

Supplementary Information.

## Data Availability

The data underlying this article will be shared on reasonable request to the corresponding author.
