# Supplementary material for: Lesion size affects the risk of technical difficulty in gastric endoscopic submucosal dissection
Source: Sci Rep. 2024 Jan 17;14:1526. doi: 10.1038/s41598-024-52150-z (PMC10794234; doi:10.1038/s41598-024-52150-z)
Supplement: Supplementary file 1 — Supplementary Information. [file 41598_2024_52150_MOESM1_ESM.docx]

**Supplementary materials**

Yuqi Zhao ^1,2,3^, Xiaogao Pan ^4,5^, Yihan Chen ^1,2,3^, Yuyong Tan ^1,2,3^ , Deliang Liu ^1,2,3^

^1^ Department of Gastroenterology of the Second Xiangya Hospital of Central South University, Changsha, China.

^2^ Research Center of Digestive Disease of Central South University, Changsha, China.

^3^ Clinical Research Center For Digestive Disease In Hunan Province, Changsha, China.

^4^ Department of Emergency Medicine of the Second Xiangya Hospital of Central South University, Changsha, China.

^5^ Emergency Medicine and Difficult Diseases Institute of Central South University, Changsha, China.

**Table S1. Baseline characteristics of the patients based on the tertiles lesion size**

| **Characteristics** | **T1 (04-15 mm)** | **T2 (15-25 mm)** | **T3 (25-70 mm)** | ***P-value*** |
| --- | --- | --- | --- | --- |
| No. patients | 121 | 104 | 180 |  |
| **Age, year** | 46.56 ± 10.12 | 50.23 ± 12.15 | 55.73 ± 11.96 | *< 0.001* |
| < 60 | 111 (91.76%) | 58 (55.78%) | 106 (58.89%) | *< 0.001* |
| ≥ 60 | 10 (8.24%) | 46 (44.23%) | 74 (41.11%) | *< 0.001* |
| **Gender** |  |  |  | *0.407* |
| male | 50 (41.32%) | 53 (50.96%) | 141 (78.33%) |  |
| female | 71 (58.68%) | 51 (49.04%) | 39 (21.67%) |  |
| **Histology** |  |  |  |  |
| epithelial lesions | 70 (57.85%) | 63 (60.57%) | 83 (65.87%) | *0.454* |
| negative for neoplasia | 34 (28.01%) | 27 (26.95%) | 26 (20.63%) | *0.416* |
| indefinite for dysplasia | 0 (0.00%) | 3 (2.88%) | 2 (1.59%) | *0.286* |
| low-grade dysplasia | 19 (15.70%) | 17 (16.35%) | 21 (16.67%) | *0.963* |
| high-grade dysplasia | 17 (14.05%) | 14 (13.46%) | 34 (26.98%) | *0.023* |
| invasive neoplasia | 0 (0.00%) | 2(1.92%) | 0 (0.00%) | *0.157* |
| non-epithelial lesions | 51 (42.15%) | 41 (39.42%) | 61 (33.89%) | *0.154* |
| **Location** |  |  |  |  |
| upper third | 21 (17.36%) | 13 (12.50%) | 26 (14.44%) | *0.629* |
| middle third | 10 (8.26%) | 14 (13.46%) | 31 (17.22%) | *0.162* |
| lower third | 90 (74.38%) | 77 (73.04%) | 123 (68.33%) | *0.561* |
| **Gross type** |  |  |  |  |
| elevated | 112 (92.56%) | 93 (89.42%) | 109 (86.51%) | *0.340* |
| flat | 7 (5.79%) | 9 (8.65%) | 14 (11.11%) | *0.413* |
| depressed | 2 (1.65%) | 2 (2.74%) | 3 (2.38%) | *0.770* |
| **Surface configuration** |  |  |  |  |
| erythema | 10 (11.76%) | 12 (16.44%) | 39 (21.67%) | *0.186* |
| ulcer | 10 (11.76%) | 8 (10.96%) | 27 (15.00%) | *0.649* |
| nodularity | 7 (8.24%) | 7 (6.73%) | 26 (14.45%) | *0.183* |
| **Submucosal fibrosis** |  |  |  | *0.451* |
| no | 117 (96.69%) | 100 (96.15%) | 167 (92.78%) |  |
| yes | 4 (3.31%) | 4 (3.85%) | 13 (7.22%) |  |
| **Invasion depth** |  |  |  | *0.964* |
| mucosa | 75 (61.98%) | 63 (60.58%) | 110 (61.11%) |  |
| non-mucosa | 46 (38.02%) | 41 (39.42%) | 70 (38.89%) |  |
| **Difficult ESD** |  |  |  | *< 0.001* |
| no | 118 (97.52%) | 90 (86.54%) | 137(76.11%) |  |
| yes | 3 (2.48%) | 14 (13.46%) | 43 (23.89%) |  |
| Procedure time, min | 48.05 ± 21.90 | 56.53 ± 31.46 | 79.73 ± 47.06 | *< 0.001* |
| Lesion size, mm | 10.33 ± 1.99 | 15.58 ± 1.15 | 28.20 ± 10.04 | *< 0.001* |

Data are presented as n (%) or mean ± standard deviation. **Abbreviations:** ESD, endoscopic submucosal dissection. The *p <0.05* is considered to be statistically significant.

**Table S2. Baseline characteristics of the patients based on the lesion location**

| Characteristics | **Upper third** | **Middle third** | **Lower third** | ***P-value*** |
| --- | --- | --- | --- | --- |
| No. patients | 60 | 56 | 289 |  |
| **Age, year** | 52.60 ± 8.73 | 52.62 ± 11.69 | 51.16 ± 12.81 | *0.665* |
| < 60 | 50 (83.33%) | 39 (69.64%) | 209 (72.32%) | *0.276* |
| ≥ 60 | 10 (16.67%) | 17 (30.36%) | 80 (27.68%) | *0.276* |
| **Gender** |  |  |  | *0.407* |
| male | 30 (50.00%) | 22 (39.29%) | 140 (48.44%) |  |
| female | 30 (50.00%) | 34 (60.71%) | 149 (51.56%) |  |
| **Histology** |  |  |  |  |
| epithelial lesions | 24 (40.00%) | 26 (46.43%) | 201 (69.55%) | *< 0.001* |
| negative for neoplasia | 12 (20.00%) | 13 (23.21%) | 73 (25.26%) | *0.653* |
| indefinite for dysplasia | 1 (1.67%) | 1 (1.79%) | 4 (1.38%) | *0.941* |
| low-grade dysplasia | 6 (10.00%) | 6 (10.71%) | 54 (18.69%) | *0.188* |
| high-grade dysplasia | 6 (10.00%) | 7 (12.50%) | 67 (23.18%) | *0.066* |
| invasive neoplasia | 0 (0.00%) | 0 (0.00%) | 1 (0.34%) | *1.000* |
| non-epithelial lesions | 36 (60.00%) | 30 (53.57%) | 88 (30.45%) | *< 0.001* |
| **Gross type** |  |  |  |  |
| elevated | 57 (95.00%) | 47 (83.93%) | 256 (88.58%) | *0.290* |
| flat | 4 (6.67%) | 4 (7.14%) | 27 (9.34%) | *0.221* |
| depressed | 1 (1.67%) | 1 (1.79%) | 8 (2.77%) | *0.234* |
| **Surface configuration** |  |  |  |  |
| erythema | 3 (5.00%) | 7 (12.50%) | 60 (20.76%) | *0.033* |
| ulcer | 6 (10.00%) | 6 (10.71%) | 41 (14.18%) | *0.065* |
| nodularity | 6 (10.00%) | 6 (10.71%) | 31 (10.73%) | *0.967* |
| **Submucosal fibrosis** |  |  |  | *0.984* |
| no | 57 (95.00%) | 53 (94.64%) | 274 (94.81%) |  |
| yes | 3 (5.00%) | 3 (5.37%) | 15 (5.19%) |  |
| **Invasion depth** |  |  |  | *0.295* |
| mucosa | 31 (51.67%) | 32 (57.14%) | 185 (64.01%) |  |
| non-mucosa | 29 (48.33%) | 24 (42.86%) | 104 (35.99%) |  |
| **Difficult ESD** |  |  |  | *0.118* |
| no | 53 (88.33%) | 42 (75.00%) | 250 (86.51%) |  |
| yes | 7 (11.67%) | 14 (25.00%) | 39 (13.49%) |  |
| Procedure time, min | 57.24 ± 39.18 | 73.85 ± 47.87 | 63.91 ± 37.86 | *0.087* |
| Lesion size, mm | 19.00 ± 10.50 | 23.21 ± 13.41 | 19.04 ± 9.67 | *0.067* |

Data are presented as n (%) or mean ± standard deviation. **Abbreviations:** ESD, endoscopic submucosal dissection. The *p <0.05* is considered to be statistically significant.

**Table S3. Baseline characteristics of the patients based on the lesion submucosal fibrosis**

| **Characteristics** | **Total** | **Non-Submucosal fibrosis** | **Submucosal fibrosis** | ***P-value*** |
| --- | --- | --- | --- | --- |
| No. patients | 405 | 384 | 21 |  |
| **Age (year)** | 51.57 ± 12.12 | 51.71 ± 12.11 | 49.13 ± 12.52 | *0.436* |
| < 60 | 298(73.58%) | 281 (73.18%) | 17 (80.95%) | *0.563* |
| ≥ 60 | 107(26.42%) | 103 (26.82%) | 4 (19.05%) | *0.563* |
| **Gender** |  |  |  | *0.270* |
| male | 191(47.16%) | 184 (47.92%) | 7 (33.33%) |  |
| female | 214(52.84%) | 200 (52.08%) | 14 (66.67%) |  |
| **Histology** |  |  |  |  |
| epithelial lesions | 251 (61.97%) | 240 (62.50%) | 11 (52.38%) | *0.479* |
| negative for neoplasia | 98 (24.20%) | 91 (23.70%) | 7 (33.33%) | *0.402* |
| indefinite for dysplasia | 6 (1.48%) | 6 (1.56%) | 0 (0.00%) | *1.000* |
| low-grade dysplasia | 66 (16.30%) | 66 (17.19%) | 0 (0.00%) | *0.080* |
| high-grade dysplasia | 80 (19.75%) | 76 (19.79%) | 4 (19.05%) | *0.978* |
| invasive neoplasia | 1 (0.25%) | 1 (0.26%) | 0 (0.00%) | *1.000* |
| non-epithelial lesions | 154 (38.02%) | 144 (37.50%) | 10 (46.62%) | *0.479* |
| **Location** |  |  |  |  |
| upper third | 60 (14.81%) | 57 (14.84%) | 3 (14.28%) | *0.933* |
| middle third | 56 (13.83%) | 53 (13.80%) | 3 (14.29%) | *0.863* |
| lower third | 289 (71.36%) | 274 (71.35%) | 15 (71.43%) | *0.980* |
| **Gross type** |  |  |  |  |
| elevated | 360 (89.89%) | 343 (89.32%) | 17 (80.95%) | *0.458* |
| flat | 35 (8.64%) | 33 (8.59%) | 2 (9.52%) | *0.525* |
| depressed | 10 (2.47%) | 9 (2.34%) | 1 (4.76%) | *0.248* |
| **Surface configuration** |  |  |  |  |
| erythema | 70 (17.28%) | 66 (17.18%) | 4 (19.05%) | *0.121* |
| ulcer | 53 (13.09%) | 49 (12.76%) | 4 (19.05%) | *0.090* |
| nodularity | 43 (10.62%) | 39 (10.16%) | 4 (19.05%) | *0.222* |
| **Invasion depth** |  |  |  | *0.082* |
| mucosa | 248 (61.23%) | 240 (62.50%) | 8 (38.10%) |  |
| non-mucosa | 157 (38.77%) | 144 (37.50%) | 13 (61.90%) |  |
| **Difficult ESD** |  |  |  | *0.038* |
| no | 345 (85.19%) | 331 (86.20%) | 12 (57.14%) |  |
| yes | 60 (14.81%) | 53 (13.80%) | 7 (33.33%) |  |
| Procedure time (min) | 64.29 ± 39.67 | 62.70 ± 39.29 | 92.67 ± 36.64 | *<0.001* |
| Lesion size (mm) | 19.61 ± 10.44 | 19.46 ± 10.49 | 22.13 ± 9.31 | *0.159* |

Data are presented as n (%) or mean ± standard deviation. **Abbreviations:** ESD, endoscopic submucosal dissection. The *p <0.05* is considered to be statistically significant.


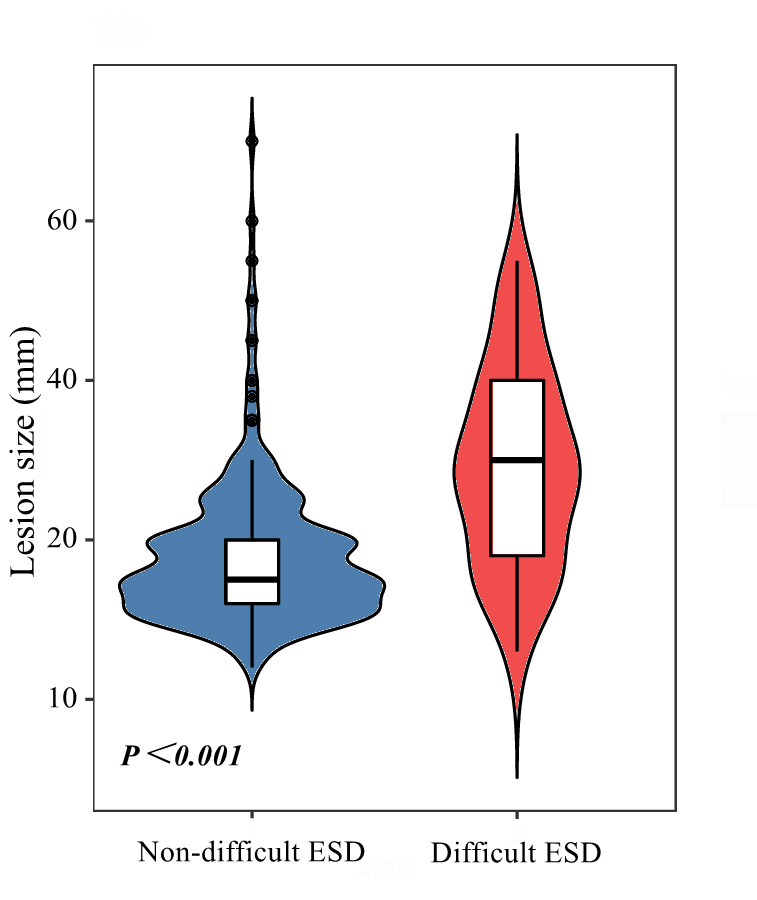


**Figure S1**. Comparison of lesion size stratified by difficult ESD


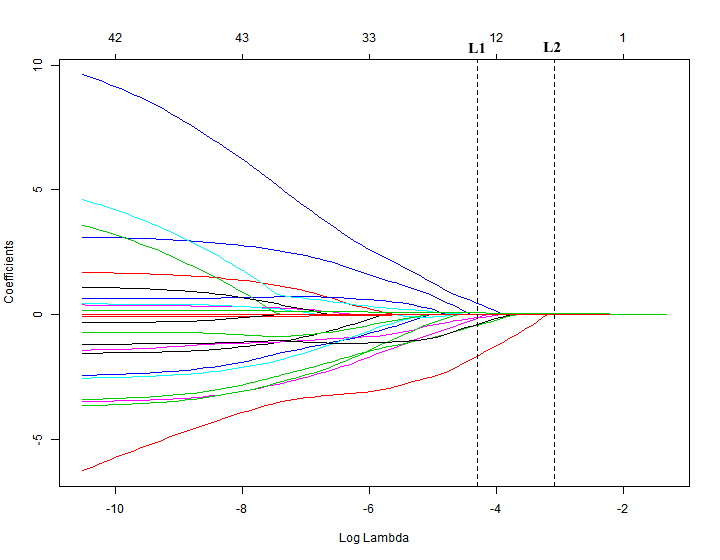

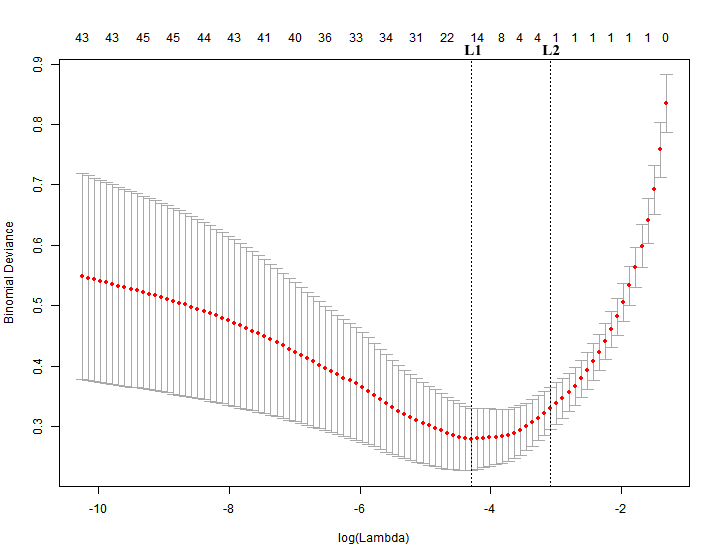


1. (B)

**Figure S2**. Feature selection using the least absolute shrinkage and selection operator (LASSO) binary logistic regression model. (A) LASSO uses the L1 penalty to select stronger predictors of the outcome while shrinking the coefficient for weaker predictors towards zero. (B) Tuning parameter (λ) selection in the LASSO model used 10-fold cross-validation via minimum criteria. (Folds for cross-validation were sampled on the person-level and stratified by event status)
